# Farmers’ Knowledge, Attitudes, and Perceptions Regarding Carcinogenic Pesticides in Fez Meknes Region (Morocco)

**DOI:** 10.3390/ijerph182010879

**Published:** 2021-10-16

**Authors:** Zineb Ben Khadda, Mustapha Fagroud, Yahya El Karmoudi, Said Ezrari, Imane Berni, Marc De Broe, Tapan Behl, Simona Gabriela Bungau, Tarik Sqalli Houssaini

**Affiliations:** 1 Laboratory of Epidemiology and Research in Health Sciences, Faculty of Medicine and Pharmacy, Sidi Mohammed Ben Abdellah University, Fez 30050, Morocco; Tarik.sqalli@usmba.ac.ma; 2 Faculty of Science and Technology, Sidi Mohammed Ben Abdellah University, Fez 30050, Morocco; Said.ezrari@usmba.ac.ma; 3 Department of Agronomy, National School of Agriculture, Meknes 50001, Morocco; mfagroud@gmail.com; 4 Laboratory of Ecology, Biodiversity and Environment, Faculty of Sciences, Abdelmalek Essaâdi University, Tetouan 93000, Morocco; yahyaelkarmoudi@gmail.com; 5 Laboratory of Functional Ecology and Engineering Environment, Department of Biology, Sidi Mohamed Ben Abdellah University, Fez 30050, Morocco; 6 Cluster of Competencies “Health and Environment”, Moulay Ismail University, Meknes 50050, Morocco; imane.berni@gmail.com; 7 Laboratory of Pathophysiology, University of Antwerp, 2000 Antwerp, Belgium; marc.debroe@uantwerpen.be; 8 Chitkara College of Pharmacy, Chitkara University, Punjab 140401, India; tapanbehl31@gmail.com; 9 Department of Pharmacy, Faculty of Medicine and Pharmacy, University of Oradea, 410028 Oradea, Romania; 10 Faculty of Medicine and Pharmacy, Doctoral School of Biological and Biomedical Sciences, University of Oradea, 410073 Oradea, Romania; 11 Department of Nephrology, University of Hospital Hassan II, Fez 30050, Morocco

**Keywords:** farmer, survey, knowledge, behavior, carcinogenic pesticides, personal protection equipment, Morocco

## Abstract

Pesticides play an important role in the improvement of agricultural production, but their use may result in adverse effects on the environment, consumers, and farmers’ health. As there are limited data focusing on the factors influencing safety behavior toward pesticide use in Morocco, we conducted a cross-sectional survey in 15 rural communities of Morocco’s Fes Meknes region to assess the attitudes, knowledge, and practices regarding pesticide use. A structured questionnaire was completed, containing the data of the interviewed farmers, their behavior towards safety measures, the type of active ingredient used, as well as the perception of risks to their own health following exposure to pesticides by the existence of chronic, self-perceived symptoms. Non-probability (empirical) sampling with the quota method was carried out, which consists of constructing the sample. Results showed that most respondents have not been trained in the application of pesticides, with almost half of the farmers using a category of pesticides which are classified by the International Agency for Research on Cancer as probable human carcinogenic (i.e., Glyphosate, Malathion). In terms of pesticide storage, 40% of farmers said that they did not store pesticides in a separate room after purchasing or using them. The empty containers were buried or burnt by half of the responders, while the remainder were thrown at the edge of fields or in public dumps. Although the participants were aware of the negative effects on their own health and on the environment caused by the application of pesticides in use, the protection measures by individual equipment were insufficient. A canonical analysis indicates that these behaviors were influenced by the farming experience, the benefit of the agricultural council services, the follow-up of training, and the education level. These variables are important factors in explaining and understanding the dangers to both the environment and health caused by pesticides. The most recorded likely consequences of pesticide exposure were visual impairment (46%), followed by dizziness (44.3%), headache (39.4%), and excessive sweating (34.4%), and 30.2% of participants identified consequent respiratory problems. Extension services targeted at safety and protection measures should be developed and accompanied by educational programs to put farmers’ perceptions into practice and encourage them to adopt healthy and environmentally friendly behaviors.

## 1. Introduction

Pesticides are widely used in agricultural production to boost productivity and quality, as well as to reduce losses caused by the attacks of different biotic factors, pests, and crop diseases [1]. However, the misuse and non-compliance with registered concentrations can have harmful consequences for both the environment and human health. Nonetheless, the excessive use of these chemicals affects the entire ecosystem by influencing food chain actors and polluting soil, groundwater, and surface water [2,3,4,5,6,7]. Humans are exposed to pesticides through various routes such as inhalation, ingestion, and dermal contact. Exposure to these harmful substances which are present in almost all environmental media (soil, water, and air) [8,9,10,11] and in the majority of food [12] can cause acute and chronic health problems. Among the many diseases that can occur, they can be listed certain cancers [13,14,15], endocrine disorders [16], abnormal reproduction [17], reduced mental capacity [18], neurodegenerative diseases [19], and modification of DNA [20]. In addition to moderate risks which include influenza, mild headaches, and blurred vision [21]. Developing countries use only 20% of worldwide pesticides, yet they undergo 99% of deaths due to pesticide poisoning [22]. In 2015, the Poison Control Moroccan Center (CAPM) established 1451 cases of pesticide poisoning which occupied the 4th position among the causes of poisoning on a national scale and still carry an increased risk for the consumer [23]. According to the World Health Organization (WHO), the annual number of poisonings is between 1 and 5 million, the majority of the most sensitive deaths of which are the elderly, infants, and children [24]. The prevalence of poverty and illiteracy in most farming areas in developing countries, including Morocco, is a major source of pesticide safety concerns [25].

Unsafe farming practices such as lack of respect for the recommended concentrations while applying pesticides and inadequate use of protective equipment were directly related to occupational poisoning rates and environmental pollution [26]. In Morocco, skin irritation caused by exposure to pesticides was due to the lack of personal protective equipment (PPE) [27]. A study in Iran [28] also found that almost half of farmers (49.5%) practiced unsafe behavior regarding the use of PPE. Similarly, less than 11% in Costa Rica and less than 2% in Uganda reported using PPE whenever handling or applying pesticides [29].

Incorrect perceptions, as well as a lack of knowledge and education [30,31] among farmers, have been identified as some of the key causes for pesticide application safety precautions not being implemented. In Ethiopia, the high rates of incorrect pesticide use were primarily due to farmers’ lack of awareness about pesticide toxicity [32]. In India, farm workers who thought pesticide application posed a high danger displayed more safety habits than other farmers, such as not smoking while handling pesticides and showering after spraying [33]. Farmers who had previously experienced spraying-related health problems were also more inclined to take precautions measures such as wearing gloves [34].

Previous research has shown that the knowledge and attitudes of farmers in agricultural settings, concerning the use of pesticides, as well as the assessment of risks associated with exposure have not been sufficiently documented in Morocco. Our study was conducted in this context, to explore the main model of active ingredients used by farmers in rural communities in the Fez-Meknes region (one of the most important agricultural regions in Morocco), to identify gaps in their knowledge, as well as to observe farmers’ awareness of the dangers to health and the environment regarding the use of pesticides. The results provide some basic information that are needed to choose the optimal path in wishing developing appropriate and sustainable pesticide management strategies, both by policy makers and researchers.

## 2. Materials and Methods

### 2.1. Study Area

The Fez-Meknes region is one of the twelve new regions of Morocco established by the territorial division of 2015. According to the 2014 General Census of Population and Habitat (RGPH), the Region has 4,236,892 inhabitants, the equivalent of about 13% of the country’s total population [35]. It covers an area of 40,075 km^2^, representing 5.7% of the Kingdom’s area. The region is known for a climate ranging from the Mediterranean to the mainland with cold winters and hot summers. The average annual precipitation varies from province to province and from season to season. As well as it enjoys a privileged location, both for surface water and groundwater. The agricultural sector is one of the promising sectors in the region. Indeed, the useful agricultural area is estimated at 1,335,639 hectares or 15% of the national useful agricultural area. The total area of irrigated land is 1,251,456 hectares, or 9% of the total area of agricultural land in the region [36].

**Figure 1:**
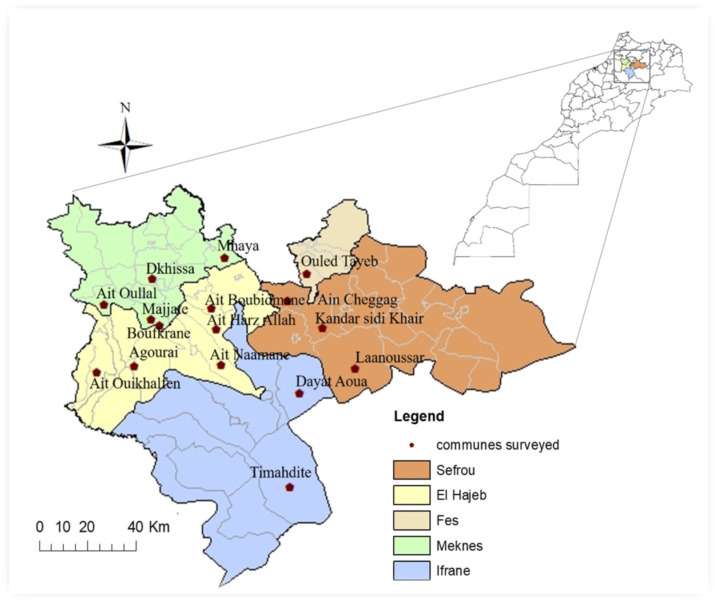
Location of the studied area.

We carried out a cross-sectional study among farmers from 15 rural communities in five provinces of the Fez Meknes region located in the northeast of Morocco: Sefrou, El Hajeb, Ifrane, Meknes, and Fez (Table 1) (specifically rural municipalities: Boufkrane, Mhaya, Majjat, Ait Ouallal, Dkhissa, Ait Naamane, Ait Boubidmane, Agourai, Ait Hraz Allah, Ait Ouikhalfen, Timhdite, Dayat Aoua, Oulad Tayeb, Laanousser, Kandar Sidi Khiar, and Ain Cheggag, as they are mentioned in Figure 1). These areas are known for significant agricultural activity and intense use of fertilizers and phytosanitary products. The sites in which the study was conducted were taken from the crop production summary report data introduced by the Regional Directorate of Agriculture in the region.

### 2.2. Sampling Method

Due to the unavailability of a list of all units in the study population, non-probability (empirical) sampling with the quota method was carried out, which consists in constructing a sample which is a reduced model of the studied population [37,38]. The inclusion of any individual farmers (who agreed to complete the survey) in the study was done by respecting the distribution of farms according to the size of the utilized agricultural area (UAA) in the Fez-Meknes region (according to Table S1). Thus, the distribution of the 526 subjects included in the study was proportional to the number of agricultural areas used. The survey was conducted door-to-door until the required number for each category was reached.

### 2.3. Data Collection

The survey took place from January to May 2019, using a pre-tested questionnaire. Respondents were not pre-informed to avoid biased responses and give them a real idea of agricultural practices. However, at the time of the interview, farmers were informed about the purpose of the study.

The questionnaire’s target items were designed based on published literature and the researchers’ previous experience in the field from past projects [34,39,40]. A review panel consisting of two specialists at the National School of agriculture in Meknes was formed to assess the research instrument. The questionnaire was piloted on a small sample of farmers prior to the study (*n* = 25). Data from this pilot sample were not used in the subsequent analysis. Then, the questions’ clarity and appropriateness were evaluated, and the questionnaire was edited accordingly. The target respondents were selected based on the following criteria: working as a farmer, >18 years old, and either the farmer owner or pesticide applicator. The surveyed farmers answered a structured questionnaire comprising objective questions (yes/no or multiple choice) and subjective questions. Group discussions were held with farmers who benefited from agricultural advisory services to obtain views on the services and training presented. As after the assessment of farmers’ hygiene behavior and practices, discussions were launched to make respondents aware of the vital importance of complying with personal protection standards at all stages of pesticide use and, at the same time, to find out why hygiene and measures for the use of PPE have not been adopted correctly.

The questionnaire was divided into four main sections:

- The first section dealt with socio-demographic issues (age, school level, family situation, a follow-up to training, and years of experience in agriculture).
- The second section focused on the phytosanitary assessment (crops and active ingredients used) and cancer risk.
- The third part was devoted to knowledge and behavior of farmers toward the use of pesticides, and the decision-making mechanisms relating to the use of phytosanitary products (the choice of active ingredients, concentration, and date of treatment), and the practices used for pesticide storage and elimination (the provision of a room fitted out for the storage of pesticides, compliance with the recommended used concentrations, the future packaging of pesticides and rinsing water from the sprayer after use). The individual protection measures taken by farmers during spraying (use of waterproof gloves, hat, boots, mask with filter cartridge, glasses, etc.), the consumption of food and drinks during the treatment, and the actions taken after application of pesticides, cleaning of clothes) were also examined.
- The last part of the questionnaire consecrated to the knowledge about the awareness and risk of pesticides to human health and the environment. Farmers were asked to select the adverse health effects experienced during or after exposure (short and/or long-term). These 10 symptoms were the most reported by farmers in several studies [41,42], as follows: dizziness, headache, excessive sweating, blurred vision, hands tremor, convulsion, loss of balance, excessive salivation, nausea/vomiting, and respiratory problems.

Interviews were conducted in the local language and verbal consent was obtained from all participants. Validation of answers on the denomination of pesticides used by the participants was confirmed in each area studied by contacting the local pesticide retailers.

### 2.4. Data Treatment

The raw data collected was reviewed after the interviews. The responses of each farmer were coded, entered, and verified to eliminate the risk of error, and then the SPSS 20 software was used to calculate the relative frequencies and frequencies of the responses. A map showing the location of the study area was prepared using ArcGIS software 10.3.1. A canonical correspondence analysis was performed to explore the strength and nature of the association between farmers’ socioeconomic characteristics (Age, Educational level, Family situation, Agricultural experience, Benefit from agricultural advisory services, Internships/Training), personal protective behaviors related to pesticide use and the perception of pesticide risks on the environment and human health. Canonical correlation provides a statistical analysis where each subject is measured on two sets of variables so as to determine if and how the two sets relate to each other [43]. Use of canonical correlation for this study enabled a more in-depth analysis of the relationship between socioeconomic variables-farmers’ protection measures and socioeconomic variables- perception of pesticide risks than would have been possible with univariate statistical procedures such as multiple regression [44].

Farmers’ protection measures have been separated into two groups to make the analysis easier to understand: the first kind (designated as behavior 1) contains personal hygiene measurements while spraying (drink and eat) and after spraying (take a shower and cleaning of clothes); the second category (described as behavior 2) is concerned with the usage of PPE such as boot masks, waterproof gloves, protective eyewear, and other similar items. Canonical correspondence analysis was carried out using the XLStat 2014.

## 3. Results

### 3.1. Descriptive Information of the Sample

The demographic characteristics of the farmers surveyed in the five provinces of the Fez Meknes region are described in Table 2. All the participants were men. The average age of the farmers was 45.02 ± 0.41 years, with 35.2% of the respondents between 41 and 50 years old, 23% between 51 and 60 years old and 6.7% under 30 years of age. Concerning education level, 43.3% of farmers surveyed had no academic training, 29.1% had primary education, 5.9% had secondary education and only 1% had university level. Most farmers were married (75.3%). A significant portion of farmers (88%) had never participated in training or internships on the application of pesticides. The average farm experience of farmers was 11.5 ± 0.16 years. Although, 80.6% of the respondents had not benefited from agricultural advisory services concerning pesticide use practices and management.

### 3.2. Phytosanitary Assessment and Cancer Risk

The population included in this study was using and was exposed to many pesticides (Table 3). In total, 40 active ingredients of commercial pesticides (with different chemical compositions) were identified during the investigation period. The most used formulations were: Deltamethrin (57.2%), Carbendazim (44.1%), Glyphosate (44.1%), Malathion (43.3%), Lambda-cyhalothrin (40.3%), Maneb (34.4%), Methomyl (31.9%) and Mancozeb (31.2%). The most used products were fungicides (42.5%), followed by insecticides with 35%, while herbicides ranked third place with 17.5% and the weakest portion was recorded by acaricides (5%).

The International Agency for Research on Cancer (IARC) defined the following groups, corresponding to degrees of indication of carcinogenicity for humans: Group 1—Carcinogenic to humans; Group 2A—Probably carcinogenic to humans; Group 2B—Possibly carcinogenic to humans; Group 3—Not classifiable as to its carcinogenicity to humans; Group 5—Probably not carcinogenic to humans [45]. Many of the pesticides have been classified as being possibly carcinogenic to humans as well as highly or moderately hazardous by WHO. In our study a part of pesticides used was classified as to its carcinogenicity risk in the same IARC monograph. For example, glyphosate was classified as probably carcinogenic to humans and chlorothalonil as possibly carcinogenic. Compared to the WHO toxicity classes, almost half (47.5%) of the declared pesticides were classified as moderately hazardous compounds, 22.5% were in class 3 and some (7.5%) were highly dangerous, notably Abamectin, Methomyl, and Dichlorovos.

### 3.3. Farmer Knowledge and Behavior towards Pesticides Use

Table S2 shows that the choice of the active ingredients and treatment concentration was based mainly on the information provided by the suppliers. If treatment was less effective, 30.2% of farmers tended to increase the concentration or change the product, while around 69.8% had consulted a specialist in plant protection products. Most farmers (85.6%) use only chemical methods to control insect pests, weeds, and cryptogamic diseases in their crops. Still, a small number of farmers have rarely used other methods (organic, cultural, and mechanical) for pest control. This portion of farmers first used rotation followed by biological control and finally the use of resistant varieties as pest management methods.

**Figure 2:**
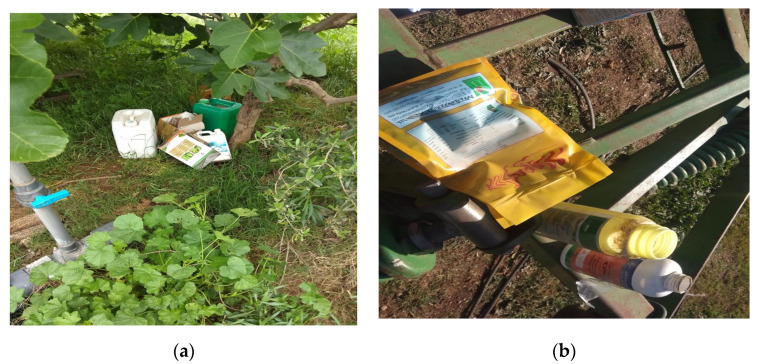
Elimination of empty pesticide packaging near a well (**a**) and at the edge of fields (**b**).

According to Table S3, it is obvious that more than half of the respondents (60.6%) had a room fitted out for the storage of phytosanitary products. The majority of farmers (83.8%) were responsible for processing by them. Most of them (72.4%) apply sprayer rinse water to treated soil after usage; while only 1% releases it into rivers. Burial (48.5%) and cremation (44.9%) were the practices most preferred by farmers to eliminate empty packaging of pesticides, a significant portion left the packaging at the edge of the fields (36.5%), and in public landfills (33.7%), and a small portion threw them into waterways (Figure 2).

PPE during and after pesticide handling are considered effective tools for reducing the risks to farmers. Most farmers, 39%, 66.2% and 48.5% of farmers, respectively, never used waterproof gloves, hats, and masks (Table S4). The boots were used by 35% of participants in each use, sometimes by 48.9%, while other items of PPE (such as masks with filter cartridges [46], and goggles) were the least considered; they were worn by a very small portion of the farmers. A look at some of the safety practices that farmers should be aware of has been conducted. Every farmer’s slurry preparation took place in the field and 76.2% of individuals said they do not eat anything while spraying, while 57 % recognized they drink something during working. After each pesticide application, most respondents said they changed and cleaned their clothes (71.1%) and had a shower (97.7%).

### 3.4. Socioeconomic Factors Influencing Farmers’ Protection Measures

**Figure 3:**
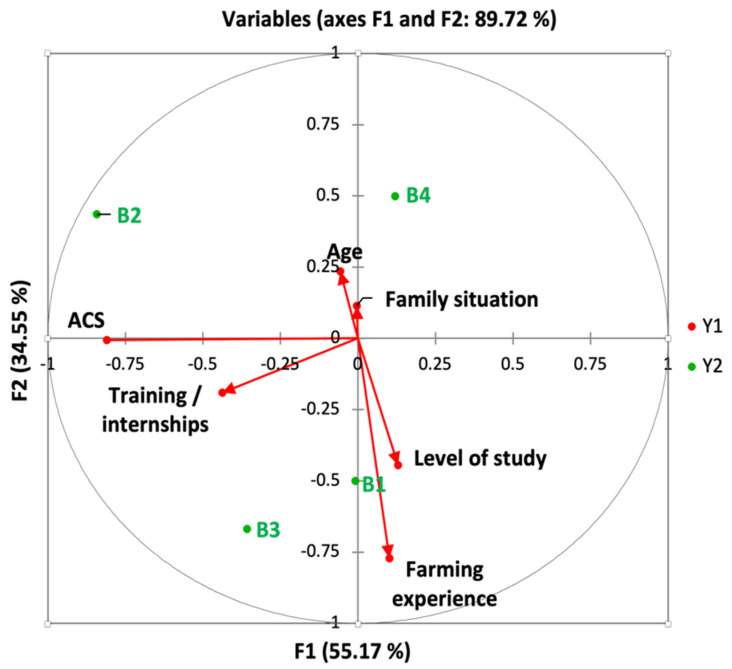
Biplot of behavior 1 and personal characteristics of the respondents’ canonical analysis constructed according to data collected. B1, drink while spraying pesticides; B2, eat while spraying; B3, shower after spraying pesticides; B4, wash clothes after spraying pesticides; ACS, benefit from agricultural advisory services. The first two canonical axes (Y1 and Y2) are in the horizontal and vertical directions. Variables of different variables of personal demographic characteristics are represented by the arrows. The arrow direction indicates the correlation between each variable and the canonical axes. The arrow length shows the relative contribution of the variables to the axes and the protective behavior-personal socioeconomic characteristics relationship, this being the principle of canonical correspondence analysis.

**Figure 4:**
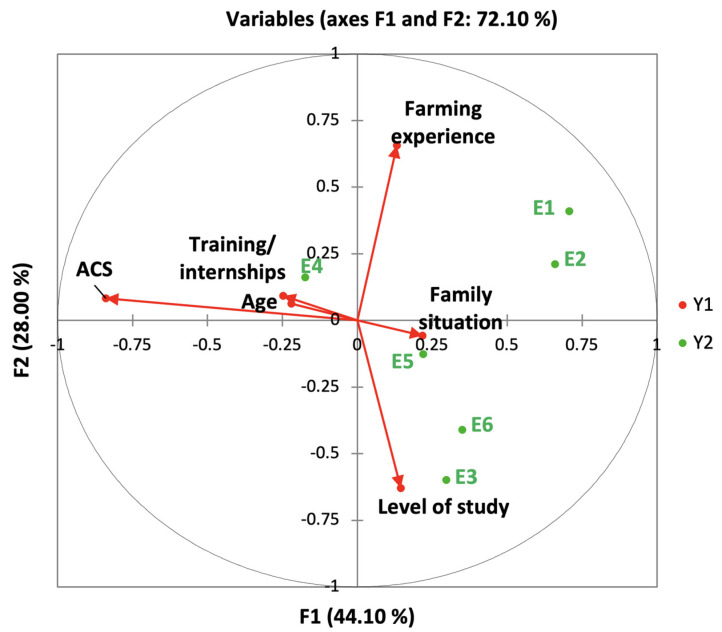
Biplot of behavior 2 and personal characteristics of the respondents’ canonical analysis as constructed according to data collected. E1, use of waterproof gloves; E2, use of a hat; E3, use of boots; E4, use of masks; E5, use of masks with cartridges; E6, use of goggles; ACS, benefit from agricultural advisory services. The first two canonical axes (Y1 and Y2) are in the horizontal and vertical directions. Variables of personal demographic characteristics are represented by the arrows. The arrow direction indicates the correlation between each variable and the canonical axes. The arrow length shows the relative contribution of the variables to the axes and the protective behavior-personal socioeconomic characteristics relationship, this being the principle of canonical correspondence analysis.

The factors impacting farmers’ protection measures were investigated using canonical correspondence analysis. These variables have been separated into two groups to make the analysis easier to understand: the first kind (designated as behavior 1) contains personal hygiene measurements, while spraying (drink and eat) and after spraying (take a shower and cleaning of clothes). The second category (described as behavior 2) is concerned with the usage of PPE such as boot masks, waterproof gloves, protective eyewear, and other similar items. Figure 3 illustrated the canonical correlation of behavior 1. According to the findings, the first canonical axes represent approximately 55.1% of the variation in behavioral characteristics, whereas the top two account for roughly 89.7%. The benefit of agricultural council services, farming experience, the educational level, and the follow-up of training and internships and have been correlated with personal hygiene behaviors (Behavior 1). Figure 4 depicts the relationship between behavior 2 and personal socioeconomic. The first canonical axes represent approximately 44.1% of the variation in the relationship between behavior 2 and socioeconomic characteristics, whereas the first two represent approximately 72.1%. The use of PPE was also linked to the three variables (The benefit of agricultural council services, farming experience, and the educational level). The other variables are not correlated with the behaviors of coverage of PPE and do not vary the percentage of explanations on the canonical axis.

### 3.5. Farmer Awareness of the Dangers of Pesticides to Human Health and Environment

**Figure 5:**
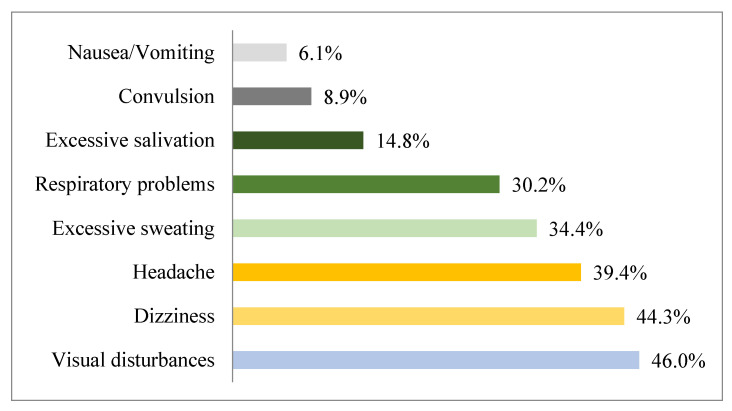
Self-reported symptoms by farmers after handling pesticides.

Farmers were asked whether they experienced these symptoms after pesticide use. The investigation of the awareness of the risks of pesticide use to the environment and human health is presented in Table S5. Pesticide residues were mentioned as risky/dangerous by 280 respondents (12.2%), while most farmers (87.8%) had no notion related to the exposure risks. This category of substances can have negative consequences on the applicators’ health, according to most participants (61.6%). The most recorded likely consequences of pesticide exposure (Figure 5) were visual impairment (46%), followed by dizziness (44.3%), headache (39.4%), and excessive sweating (34.4%). A portion of 30.2% of participants identified respiratory problems as a health effect related to pesticides. About 15% of farmers reported the problem of excessive salivation. The least reported health effects were seizures (8.9%) and nausea/vomiting (6.1%).

**Figure 6:**
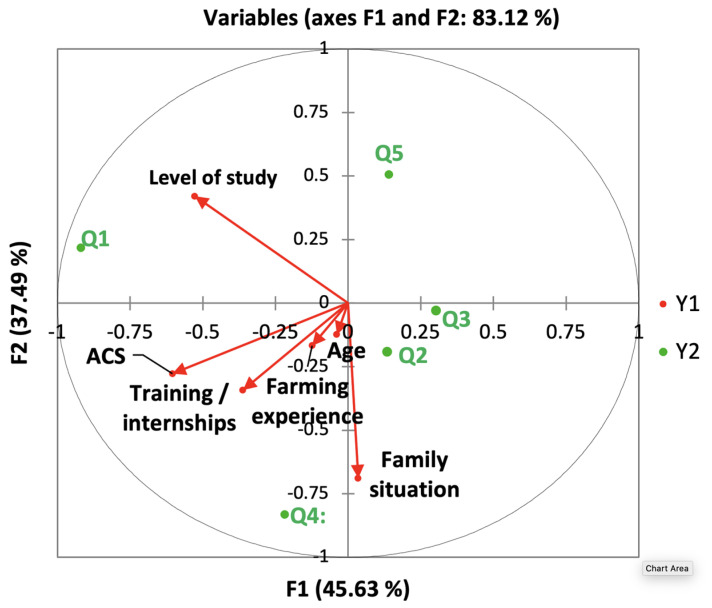
Biplot of perception of pesticide risks and social and demographic characteristics of interviewees under canonical correspondence analysis. Full questions are presented in Supplementary Materials. Q1: Do you know the pesticide residues? Q2: Do you think pesticides are harmful to human health? Q3: Did you know that the environment can be affected by pesticides? Q4: Do you think that water pollution is linked to the application of pesticides? Q5: Do you know the relationship between pesticides and disease? ACS, benefit from agricultural advisory services. The first two canonical axes (Y1 and Y2) are in the horizontal and vertical directions. Variables of different variables of personal demographic characteristics are represented by the arrows. The arrow direction indicates the correlation between each variable and the canonical axes. The arrow length shows the relative contribution of the variables to the axes and the protective behavior-personal socioeconomic characteristics relationship, this being the principle of canonical correspondence analysis.

Based on the socio-demographic characteristics of the respondents (Table 2), and the perceptions of the risks linked to pesticides (Table S5), a canonical correspondence analysis was used to reveal the factors influencing these perceptions. The perception-personal data relationship is illustrated in Figure 6. In general, the first canonical axis represents around 45.6% of the variation in the level of perception of the risk linked to pesticides and the first two represent around 83.1%. The educational level, family situation, the benefit of agricultural council services, and the follow-up of training and internships, and were correlated with the responses to the danger of pesticides (Figure 6). The location of the age and farming experience near the origin of the coordinates indicated that the effects of these risk factors on the understanding of the dangers of pesticides were similar and could not provide much explanation on the canonical axis. Consequently, education, training and internships, family situation, and the availability of agricultural advisory services were important factors in explaining the understanding the dangers of pesticides to the environment and human health.

## 4. Discussion

Nowadays, there is a growing body of evidence in research that suggests that pesticide abuse and management, as well as poor knowledge and awareness of the dangers of chemicals among farmers, has become a major issue, especially in developing countries including Morocco, which threatens the health of farmers in several ways, indeed they can be associated with the development of several serious diseases including chronic kidney disease, cancer, respiratory diseases and infertility [47,48,49]. These diseases are multifactorial and can appear after prolonged pesticide exposure. Therefore, knowing how to use pesticides and the main active ingredients used by farmers in the study area is essential. In this context, this study was conducted to assess knowledge, management strategies, and safety behaviors when handling pesticides in the Fez Meknes region, as well as their effects on health and the environment.

The farmers who took part in this study were exclusively men, which reflects the profile of farmers in rural Morocco. Previous investigations in Morocco and Pakistan revealed a similar profile [34,50]. While in some parts of China [51,52], most of the pesticide applicators were women.

The findings of this study showed that most farmers not only have a low level of education but also rely on untrustworthy sources of information when it comes to pesticide use. It has been reported that sufficient sources of information could lead to a better understanding of the risks and good management of plant protection products even for the poorly educated [53]. Indeed, most farmers have been based to choose the active ingredient and the treatment concentration based on the information provided by the suppliers. Literature indicated that commercial sellers of agrochemicals were the main sources of information regarding pest control, product selection as well as protective measures to be taken for the environment and human health [29,54].

Extensive supplier training and improved communication with farmers should be planned to help adopt rational behavior during all stages of pesticide handling. According to the data gathered, most farmers polled employed solely chemical ways to control insect pests, weeds, and pathogens in their crops, and they rarely used other pest control methods (biological, cultural, and mechanical). These findings are in line with those of a comparable survey, which found that the majority of farmers utilized chemical control of insect pests, while a negligible number of respondents used cultural (1%), organic (1%), or mechanical (2%) as a pest control method [55]. Previous studies have found that the use of this control tool can be explained by the low level of knowledge on other control measures, mainly due to farmers’ lack of access to information on integrated pest management [56], or farmers’ beliefs about the effectiveness of chemical pesticides relative to other control methods [57].

About 40% of the respondents did not store pesticides purchased in a room fitted out for phytosanitary products. This result is consistent with another study which demonstrated that most farmers have stored pesticides mainly in their homes [58], or generally in inappropriate places [59]. The storage of pesticides in areas accessible to humans and animals can increase the risk of danger and this type of behavior may be due to the lack of training of farmers on storage standards and correct methods of product management phytosanitary. Generally, poor knowledge about pesticide storage has recently been reported among farmers in other developing countries [60,61]. The immediate use of products without storing them remains a good option to mitigate the dangers of pesticides.

Most farmers apply spray rinse water after use on treated land and 26% on uncultivated land. This inappropriate application is a risky habit, many of which may be caused by this behavior, including the presence of undesirable residues in agricultural products or the persistence of harmful residues in soil. Additionally, the application of pesticides on empty land can contaminate water resources and also soil, threatening both human and animal health [6,7,62].

According to our research, half of the farmers buried or incinerated unused pesticide containers, while the other half dumped them at the edge of the fields or in public landfills. More than half of Egyptian farmers polled in research threw waste into containers [63]. Furthermore, more than 40% of Iranian respondents threw pesticide waste at the edge of the field or into irrigation canals and streams the channels [59]. On one hand, all these waste management systems are not environmentally friendly, and on the other hand, they can induce serious health concerns [64]. This is simply due to the lack of infrastructure for collecting empty containers of pesticides. As a result, training programs are aimed at educating farmers on pesticide dangers, including appropriate waste management.

Most respondents indicated that they do not drink and/or eat during treatment, so they became accustomed to taking showers and cleaning clothes regularly after applying pesticides. Generally, appropriate use and good personal hygiene are considered good practices to reduce exposure to pesticides [65], as well as a strategy to avoid poisoning after handling pesticides [66].

The use of pesticides requires protective measures to ensure the safety of sprayers. Farmers in our study seemed to be less aware of the importance of taking precautions. Almost half of the participants never wore waterproof gloves, hats, and masks. Furthermore, other PPE items such as masks with filter cartridges and goggles were the least considered by the respondents. This behavior has been recorded in several studies in developing countries, for example, most farmers in Tanzania (66.9%) said they never use PPE [41] and even in developed countries, including Brazil, a study was shown that less than 20% of farmers wore masks, waterproof clothing or gloves during pesticide spraying [67]. The educational level, the farming experience, the follow-up of training and internships, and the benefit of the agricultural council’s services are all linked to cleanliness behavior before and after spraying, as well as the usage of PPE, according to the canonical correlation analysis. These results suggest that the agricultural council’s services can encourage farmers to use pesticides responsibly by providing focused training that includes information about the importance of adhering to standards and using plant protection chemicals correctly.

The most common adverse health symptoms of pesticides were visual disturbances, dizziness, headaches, and respiratory problems. The most reported symptoms by farmers surveyed in several studies were skin irritation, headaches and flu [41], visual disturbances, extreme tiredness, headache, excessive sweating, and dizziness [53,68,69].

The survey revealed 40 active ingredients used by farmers in the region. The most frequently used are the formulations of Deltamethrin, Carbendazime, Glyphosate, Malathion, Lambda-cyhalothrin, Maneb, Methomyl, and Mancozeb. Among these 7.5% of substances are very dangerous, namely, Abamectin, Methomyl, and Dichlorovos. Similar results were found in others areas of Morocco [39], as well as in other developing countries (especially among Iranian farmers where 10% of pesticides used contained highly hazardous) [58]. IARC classified some of these pesticides such as Glyphosate, Malathion, 2,4-dichlorophenoxyacetic acid (2,4-D), and Chlorothalonil, as probable or possible human carcinogens [45,70]. A recent study has shown that in the presence of Malathion, the initiation and progression of cancer have been correlated with an increase in genomic instability and therefore the formation of tumors in animals, and signs of carcinogenesis in vitro [71]. Moreover, about 57 cancer-associated genes related to the growth and differentiation of B and T cells, immunoglobulin production, tumor suppression, and oncogenes were induced by Malathion [72].

The International Agency of the World Health Organization recently classified glyphosate as “possibly carcinogenic to humans” [73]. Although some researchers believed that glyphosate was not linked to carcinogenicity, epidemiological evidence supports a strong temporal correlation between the use of glyphosate on crops and a multitude of cancers, including breast cancer, pancreatic cancer, kidney cancer, thyroid cancer, and liver cancer [74]. The International Agency for Research on Cancer (IARC) also concluded that there is sufficient evidence in experimental animals for the carcinogenicity of glyphosate, including the appearance of several commonly occurring tumors in several tissues in rodents [75].

Exposure to the herbicide 2,4-D has in some studies been associated with an increased risk of developing non-Hodgkin-lymphoma (NHL). A meta-analysis that accounted for exposure levels, found a significantly increased risk for NHL in groups highly exposed to 2,4-D [76]. It is also associated with an increased risk of lung cancers such as small cell lung cancer (SCLC) [77].

A case-control study has been suggested that exposure to certain pesticides such as Chlorothalonil through residential proximity to agricultural applications during pregnancy may increase the risk of childhood central nervous system tumors [78]. An immunomodulatory effect of Chlorothalonil on the immune system was shown in another study, then inducing the activation of macrophages and improving the inflammatory response [79].

These pesticides used in the region of Fez Meknes, without the use of PPE and adherence to handling and enforcement standards may therefore expose farmers to a risk of uncontrolled poisoning and can cause several major public health problems, particularly chronic kidney disease of unknown origin (CKDu), which has recently been replaced by another more appropriate term; chronic interstitial nephritis in farming communities (CINAC). It mainly affects young farm workers and generally the inhabitants of agricultural areas exposed to toxic products by inhalation, ingestion of food, or consumption of contaminated water [80]. The majority of researchers confirmed that this epidemic is multifactorial, although the emphasis has been on agrochemicals from the outset [81]. In Egypt [82], where epicenters of CKDu have been recorded in rural areas, pesticides have been considered as a likely cause. Pesticides have also been suggested as a possible cause of CKDu by Indian experts [83].

Several active ingredients commonly used in phytosanitary treatment by farmers in many countries around the world have been identified as human nephrotoxins based on experimental and clinical evidence; in Sri Lanka, researchers found a strong correlation between the use of paraquat, captane, 2,4 D, and kidney damage [84]. Another case-control study [85] also confirmed that glyphosate is considered to be a metal chelating agent, and the formation of these glyphosate-metal complexes in hard water with their synergistic effects could cause kidney failure. In El Salvador, a reduction in glomerular filtration flow was recorded among farmers who used carbamate-based insecticides for the treatment of sugar cane [86].

Risk perception is an essential element in improving education, awareness, and communication systems [87]. The results of our survey show that most participants understood pesticides can have harmful effects on the health of applicators and the environment. Turkish farmers also thought that pesticides upset the balance between nature (soil, plants, and animals) and humans [88]. Similar findings have been revealed that in Ethiopia [89], Kuwait [90] and Palestine, farmers were well aware of the relationship between pesticides and health problems, including respiratory problems, while the majority of farmers (87.8%) had no idea about pesticide residues.

The canonical correlation analysis showed that the farming experience, the benefit of the agricultural council services, the follow-up of training, and the education level are correlated with personal protective behaviours and the perception of pesticide risks on the environment and human health. The results of a study conducted in Kuwait showed that education, integrated pest management training, pesticide training, information source, had significant effect on the correct use of pesticides which may help farmers take more protective behaviors, and minimize pesticide use [90]. Education and farmers’ experience can also affect the type of information sources of farmers, which in turn can influence farmers’ behavior [70]; in this regard, in a Palestinian study, significant association was reported between good level of knowledge about pesticide and education level [40]. In addition, the advice and information from government agency have been reflected in good knowledge level among the farmers [67].

These correlations suggest that the agricultural council’s services can encourage farmers to use pesticides responsibly by providing focused training that includes information about the importance of adhering to standards and using plant protection chemicals correctly. In addition, educational programs should also be implemented to improve farmers’ knowledge of the potential health and environmental risks of pesticide residues, as well as extension systems to be strengthened to put good perceptions into practice during the use of pesticides.

## 5. Conclusions

The results of our investigation show that most of the farmers were not trained in the application and management of pesticides. A total of 5% of reported products belong to the class of highly dangerous compounds. Suppliers were the main source of information in terms of choice of date, treatment concentration, and active ingredient. The chemical method was the most used average pest control. A sizeable portion of respondents revealed misuse and disposal in terms of storage and disposal of empty containers. Despite the fact that most farmers were aware of pesticide danger, measures of the use of PPE during spraying were the least considered. A canonical analysis of correspondence indicated that protection behaviors are influenced by the monitoring of training and internships, the educational level, and the benefit of agricultural council services. These last two variables, in addition to family status and farming experience, had an impact on understanding the risks of pesticides on both the environment and human health. Based on the results of this study, there is a need to improve targeted extension systems to educate farmers to comply with safety measures and standards for the correct use of pesticides. Both education and training programs should be implemented to change bad attitudes and put farmers’ perceptions into practice, and to encourage farmers to use integrated pest management and minimize the application of chemicals while keeping the best yield, by preserving health and by respecting the environment.

## Figures and Tables

**Table 1:** Characteristics of the studied areas.

Province	Altitude (m)	Latitude (North)	Longitude (West)	Climate
Meknes	552	33°53′	5°33′	Mediterranean sub-floor temperate
Fez	579	34°03′	4°58′	Mediterranean sub-floor temperate
Sefrou	823	33°49′	4°50′	Continental
El Hajeb	1000	33°41′	5°22′	Semi-arid temperate winter
Ifrane	1664	33°32′	5°06′	Mediterranean

**Table 2:** Demographic profile of the respondents (*n* = 526).

Variable	*n*	%
Age (years) (mean 45.02; SD 9.59)		
<30	35	6.7
30–40	153	29.1
41–50	185	35.2
51–60	121	23
>60	32	6.1
Farming experience (mean 11.56; SD 3.84)		
5–10	266	50.6
11–20	243	46.2
>20	17	3.2
Family situation		
Single	118	22.4
Married	396	75.3
Widower	7	1.3
Divorced	5	1
Benefit from agricultural advisory services (ACS)		
Yes	63	12
No	463	88
Training/Internship		
Yes	102	19.4
No	424	80.6
Education study		
Illiterate	288	43.3
Primary	153	29.1
College	109	20.7
Secondary	31	5.9
University	5	1

**Table 3:** Pesticides used by farmers with IARC classification and WHO toxicological class.

Name	Chemical Family	Class ^a^	IARC	*n*	%
Deltamethrin	Pyrethroids	II	3	301	57.2
Carbendazim	Benzimidazoles	U	-	232	44.1
Glyphosate	Amino phosphanates	III	2A	232	44.1
Malathion	Organophosphorus	III	2A	228	43.3
Lambda cyhalothrin	Pyrethroids	II	-	214	40.7
Maneb	Dithiocarbamates	U	3	181	34.4
Methomyl	Carbamates	Ib	-	168	31.9
Mancozeb	Dithiocarbamates	U	-	164	31.2
Abamectin	Avermectins	Ib	-	148	28.1
Dimethoate	Organophosphorus	II	-	141	26.8
Dicofol	Carbinols	II	3	136	25.9
Cypermethrin	Pyrethroids	II	-	131	24.9
Captan	Phtalimides	U	3	118	22.4
Thiophanate methyl	Benzimidazoles	III	-	104	19.8
Ziram	Dithiocarbamates	II	3	94	17.9
Paraquat	Bipyridiles	II	-	91	17.3
Sulphur	Minerals	III	-	82	15.6
Thiram	Dithiocarbamates	II	3	82	15.6
Probinebe	Dithiocarbamates	U	-	79	15
Oxyfluorfen	Diphenyl ethers	U	-	67	12.7
Thiacloprid	Chloronicotiniles	II	-	60	11.4
Dodine	Guanidine	II	-	58	11
Fluazifop-P-butyl	Aryloxy phenoxy-propionates	III	-	54	10.3
Chloropyriphos ethyl	Organophosphorus	II	-	48	9.1
Difenoconazole	Triazoles	II	-	47	8.9
Azoxystrobine	Strobilurins	U	-	44	8.4
Iprodine	Dicarboximides	III	-	42	8
2,4-D	A. phenoxy-alkanoic	II	2B	41	7.8
Propargite	Sulfites	III	-	41	7.8
Indoxacarb	Indoxacarb	II	-	36	6.8
Copper oxychloride	Minerals	II	-	43	8.1
Copper hydroxide	Minerals	II	-	30	5.7
Imidoclopride	Neonicotinoids	II	-	27	5.1
Acétamipride	Chloronicotiniles	-	-	25	.8
2,4 MCPA	A. phenoxy-alkanoic	II	-	24	4.6
Dichlorovos	A. phenoxy-alkanoic	Ib	-	16	3
Chlorothalonil	Chloronitriles	U	2B	14	2.7
Endosulfan	Triazole	II	-	9	1.7
Hexaconazole	Triazoles	III	-	8	1.5
Tau-fluvinate	A. phenoxy-alkanoic	III	-	5	1
Dicamba	Benzoic acids	II	-	4	0.8

^a^ Ib, highly hazardous; II, moderately hazardous; III, slightly hazardous; U, unlikely to pose an acute hazard in normal use. IARC, International Agency for Research on Cancer.

## Data Availability

The data that support the findings of this study are available from the first author, upon reasonable request.

## Supplementary Materials

The following are available online at https://www.mdpi.com/article/10.3390/ijerph182010879/s1, Table S1: Description of non-probability (empirical) sampling with the quota method; Table S2: Knowledge and decision-making mechanism related to pesticide use practices, Table S3: Behavior of farmers for storing and disposing of pesticides, Table S4: Precautionary measure of the farmers surveyed, Table S5: Awareness of the risks of pesticides on the environment and human health.
