# Supplementary material for: Farmers’ Knowledge, Attitudes, and Perceptions Regarding Carcinogenic Pesticides in Fez Meknes Region (Morocco)
Source: Int J Environ Res Public Health. 2021 Oct 16;18(20):10879. doi: 10.3390/ijerph182010879 (PMC8535244; doi:10.3390/ijerph182010879)
Supplement: Supplementary file 1 [file ijerph-18-10879-s001.zip › ijerph-1398305-supplementary.pdf]

International Journal of
Environmental Research and Public Health

MDPI

Supplementary Materials

Table S1. Description of non-probability (empirical) sampling with the quota method.

Parameters	Province					
	**Sefrou**	**Fès**	**Meknes**	**El Hajeb**	**Ifrane**	**Total**
Area (Ha)						
Vegetable gardening	820	3460	7032	9127	1434	21,873
Arboriculture	6670	3772	8498	15,331	462	35,733
Percentage %						
Vegetable gardening	4	16	32	42	7	100
Arboriculture	19	11	24	43	4	100
Factor						
Vegetable gardening	1	4	8	10	2	25
Arboriculture	5	3	6	10	1	25

Table S2. Knowledge and decision-making mechanism related to pesticide use practices.

No	Question	*n* (%)
1	To initiate treatment, you rely on?	
	$\chi^2 = 361.1$ **	
	Harmfulness threshold	239 (45.2%)
	The date fixed in advance	242 (46%)
	Regional surveillance	9 (1.7%)
	Neighbors	37 (7%)
2	What support is available for decision making in terms of choice of the **date** of treatment?	
	$\chi^2 = 357.1$ **	
	Harmfulness threshold	21(4%)
	The date fixed in advance	258 (49%)
	Regional surveillance	220 (41.8%)
	Neighbors	27(5.1%)
3	What support is available for decision making in terms of choice of the concentration of treatment	
	$\chi^2 = 673.1$ **	
	Harmfulness threshold	2 (0.4%)
	The date fixed in advance	366 (69.6%)
	Regional surveillance	154 (29.3%)
	Neighbors	4 (0.8%)
4	What support is available for decision making in terms of choice of the active ingredient**?**	
	$\chi^2 = 683$ **	4 (0.8%)
	Harmfulness threshold	375 (71.3%)
	The date fixed in advance	135 (25.7%)
	Regional surveillance	12 (2.3%)
	Neighbors	
5	If treatment is ineffective, will you rather tend?	
	$\chi^2 = 82.2$ **	
	increase the concentration or change the product.	159 (30.2%)
	consult a specialist in phytosanitary products	367 (69.8%)
6	Where do you buy phytosanitary products?	
	$\chi^2 = 452.7$ **	
	retailer	507 (96.4%)
	company directly	19 (3.6%)
7	Do you use other methods than chemical methods?	
	$\chi^2 = 655$ **	450 (85.6%)
	Never	67 (12.7)
	Rare	9 (1.7)
	Often	
8	If so, what are these methods?	
	$\chi^2 = 1036$ **	
	biological control	19 (25%)
	variety resistance	7 (9.2%)
	rotation	50 (65.8%)

** $p < 0.05$.

Table S3. Behavior of farmers for storing and disposing of pesticides.

No	Question	*n* (%)
1	Do you have a room fitted out for the storage of phytosanitary products?	
	$\chi^2 = 23.8$ **	
	Yes	319 (60.6%)
	No	207 (39.4%)
2	Do you respect the recommended use of concentrations?	
	$\chi^2 = 385$ **	
	Often	57 (10.8%)
	Frequent	82 (15.6%)
	Always	387(73.6)
3	Do you take into account the climate (rain, wind ...) to carry out a treatment?	
	$\chi^2 = 475$ **	
	Yes	513 (97.5%)
	No	13(2.5%)
4	Do you take into account the time of day (morning, midday, evening) when performing treatment?	
	$\chi^2 = 338$ **	
	Yes	474 (90.1%)
	No	52 (9.9%)
5	Who's in charge of treatment?	
	$\chi^2 = 596.8$ **	
	Myself	438 (83.3%)
	qualified person	20 (3.8%)
	simple farm worker	68 (12.9%)
6	What is the fate of the sprayer rinse water after its use?	
	$\chi^2 = 413$ **	
	Application in the treated field.	381 (72.4%)
	Application on uncultivated land	140 (26.6%)
	Release into waterways	5 (1%)
7	What is the fate of chemical packaging?	
	Leave at the edge of the fields	
	$\chi^2 = 41.4$ **	
	Yes	192 (36.5%)
	No	334(63.5%)
	Burial	
	$\chi^2 = 0.4$	
	Yes	255(48.5%)
	No	271 (51.5%)
	Public landfills	
	$\chi^2 = 56.2$ **	
	Yes	177 (33.7%)
	No	349 (66.3%)
	Streams	
	$\chi^2 = 212$ **	
	Yes	96 (18.3%)
	No	430 (81.7%)
	Cremation	
	$\chi^2 = 5.5$ **	
	Yes	236 (44.9%)
	No	290 (55.1%)

** $p < 0.05$.

Table S4. Precautionary measure of the farmers surveyed.

No	Question	*n* (%)
1	Use of waterproof gloves	
	$\chi^2 = 41.4$ **	
	Never	205 (39%)
	Sometimes	215 (40.9%)
	Every use	106 (20.2%)
2	Use of hat	
	$\chi^2 = 294.7$ **	
	Never	348 (66.2%)
	Sometimes	148 (28.1%)
	Every use	30 (5.7%)
3	Use of masks	
	$\chi^2 = 128.2$**	
	Never	255 (48.5%)
	Sometimes	216 (41.1%)
	Every use	55 (10.5%)
4	Use of boots	
	$\chi^2 = 90.3$ **	
	Never	80 (15.2%)
	Sometimes	257 (48.9%)
	Every use	189 (35.9%)
5	Use of mask with filter cartridge	
	$\chi^2 = 998.7$ **	
	Never	517 (98.3%)
	Sometimes	2 (0.4%)
	Every use	7 (1.3%)
6	Use of goggles	
	$\chi^2 = 419.2$ **	
	Never	385 (73.2%)
	Sometimes	132 (25.1%)
	Every use	9 (1.7%)
7	Porridge preparation place	
	Orchard	526 (100%)
8	Consume food while spraying	
	$\chi^2 = 144.8$ **	
	Yes	125(23.8%)
	No	401 (76.2%)
9	Consume drinks while spraying	
	$\chi^2 = 10.4$ **	
	Yes	300 (57%)
	No	226 (40.7%)
10	What types of measures do you take after application pesticides?	
	Take a shower	
	$\chi^2 = 479$	
	Yes	514 (97.7%)
	No	12 (2.3%)
	Cleaning clothes	
	$\chi^2 = 93.6$	
	Yes	374 (71.1%)
	No	152 (28.9%)

** $p < 0.05$.

Table S5. Awareness of the risks of pesticides on the environment and human health.

No	Question	*n* (%)
1	Do you know the pesticide residues?	
	$\chi^2 = 301.1$ **	
	Yes	64 (12.2%)
	No	462 (87. 8%)
2	Do you think pesticides are harmful to human health?	
	$\chi^2 = 605.8$ **	
	Neutral	126 (24%)
	Average for	76 (14. 4%)
	Agree	324 (61.6%)
3	Did you know that the environment can be affected by pesticides?	
	$\chi^2 = 227.5$ **	
	Yes	436 (82.9%)
	No	90 (17.1%)
4	Do you think that water pollution is linked to the application of pesticides?	
	$\chi^2 = 196.2$ **	
	Neutral	67 (12.7%)
	Average for	19 (3.6%)
	Agree	440 (83.7)
5	Do you know the relationship between pesticides and disease?	
	$\chi^2 = 90.3$ **	
	Yes	372 (70.7%)
	No	154 (29.3%)

** $p < 0.05$.
